# Biosecurity Insights from the United States Swine Health Improvement Plan: Analyzing Data to Enhance Industry Practices

**DOI:** 10.3390/ani14071134

**Published:** 2024-04-08

**Authors:** Michael Harlow, Montserrat Torremorell, Cristopher J. Rademacher, Jordan Gebhardt, Tyler Holck, Leticia C. M. Linhares, Rodger G. Main, Giovani Trevisan

**Affiliations:** 1 College of Public Health, George Mason University, Fairfax, VA 22030, USA; 2 College of Veterinary Medicine, Veterinary Diagnostic and Production Animal Medicine, Iowa State University, Ames, IA 50011, USA; 3 Department of Veterinary Population Medicine (VPM), College of Veterinary Medicine, University of Minnesota, St. Paul, MN 55108, USA; 4 Department of Diagnostic Medicine/Pathobiology, College of Veterinary Medicine, Kansas State University, Manhattan, KS 66506, USA

**Keywords:** swine health, biosecurity, foreign animal disease, animal health programs, Swine Health Improvement Plan

## Abstract

**Simple Summary:**

This article presents a snapshot of biosecurity practices implemented across the United States swine industry. Foreign animal diseases and endemic emerging and re-emerging swine diseases are serious threats. Biosecurity practices reported during enrolment in the United States Swine Health Improvement Plan are summarized here and provide insights into areas where the US Swine industry is prepared and identified opportunities and areas of opportunity for improvement in biodefense. This voluntary collaborative animal health program effort provides a baseline of biosecurity efforts over a significant portion of the swine industry. The findings can help guide the swine industry to continue to improve biosecurity, making sure the industry stays strong and sustainable despite new challenges.

**Abstract:**

Biosecurity practices aim to reduce the frequency of disease outbreaks in a farm, region, or country and play a pivotal role in fortifying the country’s pork industry against emerging threats, particularly foreign animal diseases (FADs). This article addresses the current biosecurity landscape of the US swine industry by summarizing the biosecurity practices reported by the producers through the United States Swine Health Improvement Plan (US SHIP) enrollment surveys, and it provides a general assessment of practices implemented. US SHIP is a voluntary, collaborative effort between industry, state, and federal entities regarding health certification programs for the swine industry. With 12,195 sites surveyed across 31 states, the study provides a comprehensive snapshot of current biosecurity practices. Key findings include variability by site types that have completed Secure Pork Supply plans, variability in outdoor access and presence of perimeter fencing, and diverse farm entry protocols for visitors. The data also reflect the industry’s response to the threat of FADs, exemplified by the implementation of the US SHIP in 2020. As the US SHIP program advances, these insights will guide industry stakeholders in refining biosecurity practices, fostering endemic re-emerging and FAD preparedness, and ensuring the sustainability of the swine industry in the face of evolving challenges.

## 1. Introduction

The United States of America (US) is the third largest producer and consumer of pork. The US is also the second largest pork exporter, supplying almost 30% of the world’s pork products [1]. The US pork industry creates over 600,000 jobs, producing over USD 35 billion in private income and USD 50 billion in gross national product (GDP) [2]. Endemic diseases, e.g., porcine reproductive and respiratory syndrome virus (PRRSV), pose a continuous threat, with PRRSV alone causing annual economic losses greater than USD 600 million to the US swine industry [3]. The potential introduction of a foreign animal disease (FAD) also poses a constant threat to the US swine industry. The most recent introduction of a FAD to the US swine industry occurred in 2013 with porcine epidemic diarrhea virus (PEDV) and in 2014 with porcine deltacoronavirus (PDCoV) [4]. Within 8 weeks of first PEDV diagnosis, the disease was already present in 12 US states [5] and caused the death of an estimated 5 million pigs [6], and it became endemic in the US [7].

FADs like African swine fever (ASF) have never been detected in the US, while classical swine fever (CSF) was eradicated in 1978, and foot-and-mouth disease (FMD) in 1929 [8,9]. ASF is species-specific, affecting only Suidae, and it is currently spreading throughout Europe, Asia, and Africa, significantly impacting swine trade, as it steadily creeps toward the US. The epidemiological link for the European ASF outbreak was identified as contaminated fomites on transport ships. In 2021, ASF was discovered in Haiti and Dominican Republic, which are located about a 1000 km from the US continental shores [10], or about 380 km from Puerto Rico, a US territory, making ASF one of the most eminent FAD threats to the US swine industry. ASF persists in the environment for long periods of time, has multiple routes of infection, and has high morbidity and mortality [11]. FAD agents, such as the ASF virus (ASFV), can be transported on fomites, including materials such as clothing, utensils, and farm tools [12]. Wild pig populations are susceptible to ASFV, and if wild pigs become infected, their presence could contribute to the maintenance of ASF in a region. ASF has been found to retain viability in feces and urine for up to eight days and in certain feed ingredients for up to 30 days [13]. ASFV has been found in decaying wild boar carcasses for at least 15 weeks, which serve as an environmental reservoir and important vector in maintaining the disease in wild boar populations because pigs are omnivorous [14]. Once an FAD is introduced to an area, it can be maintained in reservoirs of sylvatic pigs and can be transmissible through contact between sylvatic and domestic animals, and some FADs can be transmitted through tick vectors [12]. There are three species of soft ticks, *Ornithodoros coriaceus*, *O. turicata*, and *O. puertoricienses*, present in the US that are thought to be able to transmit ASFV [15]. The presence of wild boars in some regions of the US coupled with the presence of potential ASFV tick reservoirs can pose an additional level of complexity for control and eradication in case this FAD reaches the US continental territory.

Biosecurity includes measures and behaviors to mitigate or prevent the entrance of a given pathogen/disease or infestation in a farm or region and its subsequent establishment, to exclude it, and to reduce its spread to/from and within an animal population and reduce its impact once it has been introduced [16]. Biosecurity practices are broadly defined and include everything entering the premises, such as animals, people, supplies, and the vehicles that transport animals, feed, and maintenance materials [17]. Specific biosecurity practices can include requiring showering before entry and the usage of farm-specific clothes, as well as bench entry systems; meanwhile, biocontainment is related to the measures put in place to prevent the spread of the pathogen/disease to other farms, e.g., proper quarantining of new livestock [18,19]. Showering in and out of facilities is an effective procedure for reducing the possibility of pathogen entry into and exiting from a site through personnel. Trailers and transport vehicles are a concerning source of disease spread, as they travel from multiple locations and can carry organic matter, providing ample opportunities for disease to spread [20,21,22,23]. This includes feed delivery, as those vehicles travel to multiple farms per day and have a common point source, i.e., the feed mill. The thorough cleaning of transportation equipment is a priority in a successful biosecurity plan. On-farm biosecurity practices can be used to characterize the level of biosecurity on given farms and generate a score for practices that distinguish the biosecurity levels across sites [24] and assess the relative vulnerability of swine breeding herds to the introduction of pathogens such as porcine reproductive and respiratory syndrome virus [25].

The increase in the efficiency of pork production has resulted in the consolidation of pork producers. The reduced number of operations has resulted in larger operations with an increase in the number of heads per farm, mainly located in the US Midwest states [26,27]. These large facilities greatly improve production efficiency, but the nature of this design creates unique challenges for biosecurity. These challenges can be addressed through site-specific biosecurity plans, such as the US example of the Secure Pork Supply (SPS) (https://www.securepork.org, accessed on 5 December 2023) plans, providing a framework to create site-specific biosecurity, animal movement, and business continuity plans in the face of an FAD event. As an example, understanding who enters a site, when they visited, and where they were previously is essential to tracing the possible disease spread during a disease outbreak investigation. Epidemiologists and State and Federal Animal Health Officials can use visitor logbooks to trace back human contacts to find potential links of where an outbreak could have started. With this information, they can identify and address the issues that allowed for the entrance of the pathogen into the site and prevent further disease spread.

There is growing concern about the potential introduction of FADs into a country through contaminated feed ingredients [28,29]. Ingredients used in US swine diets are sourced globally, with some of those regions being affected by FADs, including ASF and/or CSF. Pathogens such as ASFV have been shown to potentially survive for weeks in both plant- and animal-based ingredients [30]. Ensuring the provision of safe food for the animals is a big step in FAD preparation. Ingredients originating from ASF-positive countries are particularly concerning given the extended survival characteristics of ASFV [28]. An extended feed ingredient holding time, a common biosecurity practice implemented by ingredient importers and feed manufacturers, allows for the natural decay of viruses to occur [31]. Holding times will depend on the type of feed/ingredient, ambient temperature, and environmental conditions. Additionally, there are chemical feed additives, also known as feed mitigants, which are safe for animal consumption that can be added to the diet, with experimental evidence demonstrating the potential to reduce the detection and infectivity of viruses.

Having a defined, consistent, broadly adopted, and internationally recognized biosecurity plan is paramount in mitigating the impact of an introduced FAD and helps to further enhance disease preparedness for foreign and endemic diseases. A new national health certification program for the swine industry was implemented in the US in late 2020, the US Swine Health Improvement Plan (US SHIP, https://usswinehealthimprovementplan.com/, accessed on 5 April 2023). US SHIP is a voluntary, collaborative effort between industry, state, and federal entities modeled after the National Poultry Improvement Plan (NPIP, https://www.poultryimprovement.org/default.cfm, accessed on 5 April 2023). US SHIP has been specifically organized to meet the unique needs and challenges of the U.S. swine industry. US SHIP is able to use the lessons learned by the NPIP over the last 80-plus years to fast-track a plan that addresses the concerns of the swine industry to prevent the introduction of trade impacting diseases such as ASF and CSF, as well as respond to and mitigate their impact if found in the US.

US SHIP is creating an industry-led effort to protect animal health, mitigate the impact of an FAD on the economy, and have a sustainable process to re-establish trade from unaffected sites outside of control zones. US SHIP is a new initiative to support FAD preparedness, with an emphasis on the demonstration of the freedom of disease outside of control zones, creating a solid basis for FAD preparedness that the United States Department of Agriculture (USDA)’s ASF Response Plan the Red Book recognizes as a new US initiative for FAD preparedness [11]. Thus, a critical step in disease preparedness is a need to understand the current state of biosecurity practices implemented by sites participating in the program. As part of US SHIP enrollment, producers are required to provide site(s) demographic(s) information, fill out a biosecurity survey, and profess their willingness to comply with program standards. The US SHIP biosecurity survey results create a snapshot of the US swine industry’s current level of biosecurity, which helps identify areas of improvement, guide future studies, and assess infrastructure building. The current study summarizes the biosecurity practices reported by the producers enrolled in the US SHIP program and provides a general assessment of practices implemented across the US swine industry.

## 2. Materials and Methods

To capture the current biosecurity practices being implemented in swine farms at the time of US SHIP enrollment, an online survey was created and distributed to participants using a commercial survey builder (Qualtrics XM, https://www.qualtrics.com/ (accessed on 5 January 2020), Seattle, WA, USA). The survey questions were crafted to obtain a broad understanding of the status of biosecurity practices being implemented in the US swine industry. All producers’ sites enrolled with US SHIP were asked to complete a biosecurity survey for all of their sites. The end goal of the biosecurity survey was to capture and benchmark the biosecurity practices implemented across the swine industry.

The survey consisted of initial screener questions to collect site demographics and the respondent’s contact information. Surveys were filled out for the production type, for each producer, (e.g., boar stud, sow farm, etc.), for each state. For example, if a producer had three breeding herds in one state and two in another state, then they would fill out two surveys in total. The initial enrollment survey questions collected site demographic information for the respective state(s) in which the site(s) were located, enabling us to capture the number of participating sites in US SHIP, categorized by site type in each state. Eight site types were listed in the survey: boar stud, breeding herd, growing pig, farrow-to-feeder/finish, small holding, non-commercial, packing plant, and live animal marketing operators. Questions include a free range of responses in percentage or Lickert-scale format (Table 1) and were sectioned by site types (Table 2). A complete set of questions is provided in the Supplementary Materials (File S1).

Survey responses were recorded in Qualtrics. An R markdown script was written using an Application Program Interface (API) connection that is available in the R package Qualtrics to connect survey responses to a wrangling and graphical application built using the R package tidyverse, which summarizes and presents results in a standardized graphical and table-content formats, providing basic survey results. Analytical and graphical displays of survey responses were performed in R version 4.3.0 (R Core Team (2023), Vienna, Austria, https://www.R-project.org, accessed on 5 April 2023).

## 3. Results

**Figure 1:**
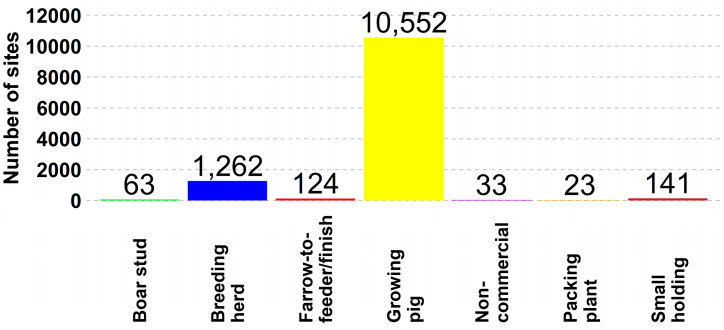
Number of sites by type of production site.

**Figure 2:**
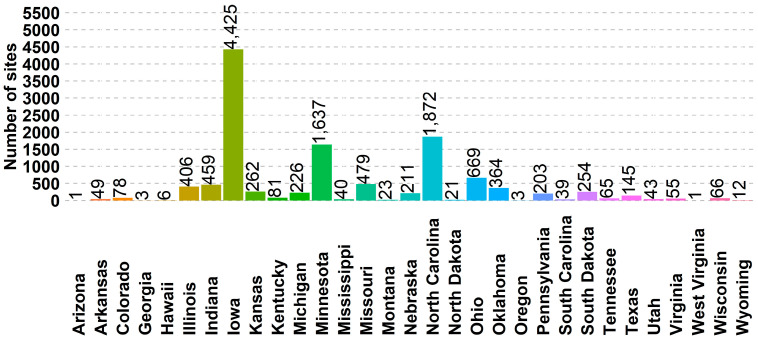
Number of sites that completed the US SHIP survey at enrollment by state.

From January 2022 to 1 March 2024, surveys were completed for 12,195 individual production sites across 31 states. Growing pigs made up over 86% (10,552 of 12,195) of the responding sites; 10% (1262 of 12,195) for breeding herds; 1% (141 of 12,195) for small holding; 1% (121 of 12,195) for farrow-to-finish; and the remaining for non-commercial, boar stud, and packing plants (Figure 1). The three states with the highest number of US SHIP-enrolled sites were Iowa, with 36% (4425 of 12,195); North Carolina, with 15% (1872 of 12,195); and Minnesota, with 13% (1637 of 12,195). The remaining 36% (4261 of 12,195) of the sites were in the other 28 states (Figure 2).

**Figure 3:**
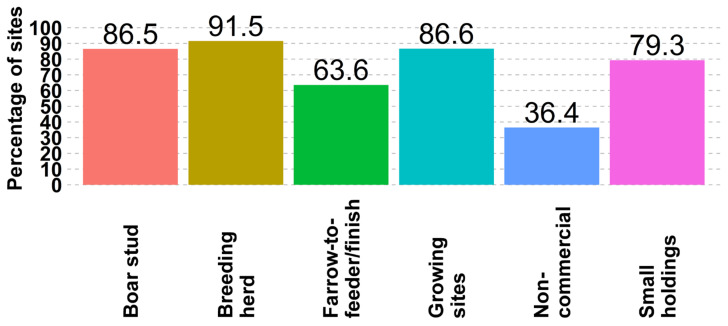
Percentage of sites by site type that have completed the Secure Pork Supply (SPS) plans.

From participant sites, 91.5% (1154 of 1262) of responding breeding herds, 87% (9149 of 10,552) of growing pigs, 87% (54 of 63) of boar studs, 36% (12 of 33) of non-commercial sites reported having a completed SPS plan in place (Figure 3). SPS plans provide an industry-acceptable base for creating credible, provable disease-free zones in the instances of an FAD introduction. These zones could be leveraged through international trade agreements to support the continuity of business.

**Figure 4:**
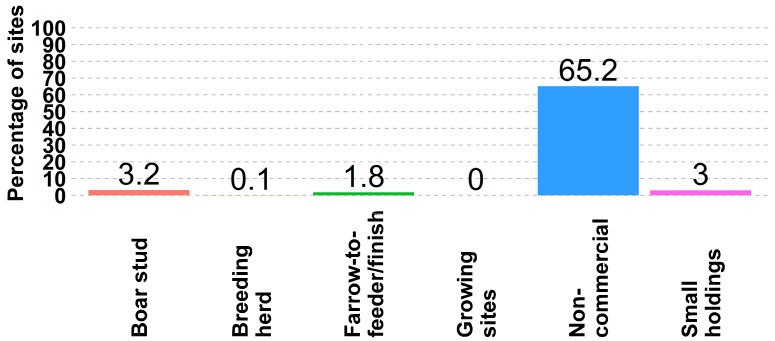
Percent of sites by site type where animals have access to the outdoors.

**Figure 5:**
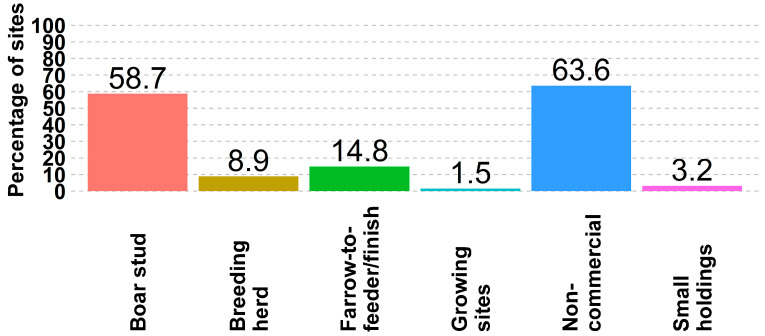
Percentage of sites, by site type, where sites have perimeter fences.

While 65% (22 of 33) of non-commercial sites allow animals to access the outdoors, 64% (21 of 33) of non-commercial sites also have perimeter fencing. While only 3.2% (2 of 63) of boar stud sites allow outdoor access, 58.7% (37 of 63) of the sites have perimeter fencing (Figure 4 and Figure 5). In general, perimeter fences are not widely adopted in the US swine industry.

**Figure 6:**
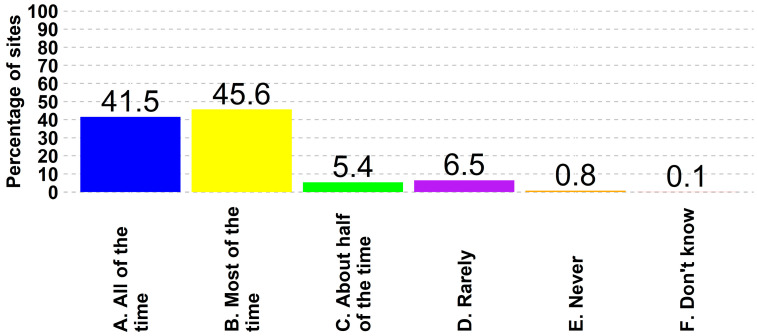
Percentage of responses by procedure frequency where visitors sign a log-in book prior to entering a site(s).

For tracking visitors on site, 42% (5051 of 12,172) of responding sites stated that they require visitors to sign a logbook all the time, another 46% (5550 of 12,172) reported doing so most of the time, and less than 1% (97 of 12,172) of all sites stated that they never require visitors to sign in. A breakdown by site type reveals that 94% (1186 of 1262) of the breeding herds reported that they require visitors to sign in via log-entry books all the time. Farrow-to-feeder had a widely dispersed response, with non-commercial sites reporting that 55% (18 of 33) of them do not have any entry log requirement (Figure 6).

**Figure 7:**
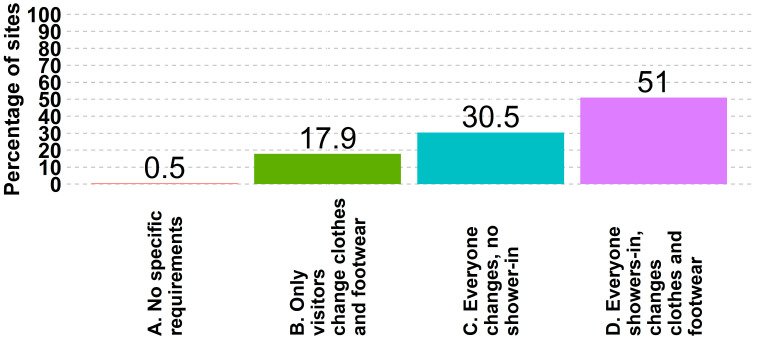
Percentage of sites that require a given procedure to enter a farm.

Of all responding sites, 51% (6219 of 12,195) require everyone to shower in and change foot and outerwear, 30% (3719 of 12,195) require everyone to change foot and outwear but not shower, 18% (2195 of 12,195) require only visitors to change clothes and footwear, and 0.5% (61 of 12,195) stated they have no entry requirements (Figure 7). Breeding herds had the largest percentage of sites requiring everyone to shower and change his/her outerwear 95% (1192 of 1262).

**Figure 8:**
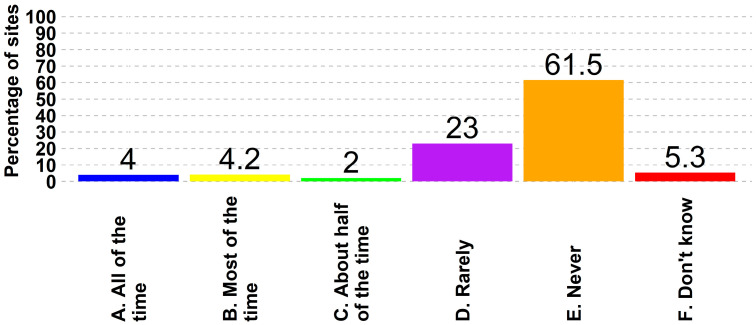
Frequency of feed mitigants used in feed rations to reduce disease transmission risk.

**Figure 9:**
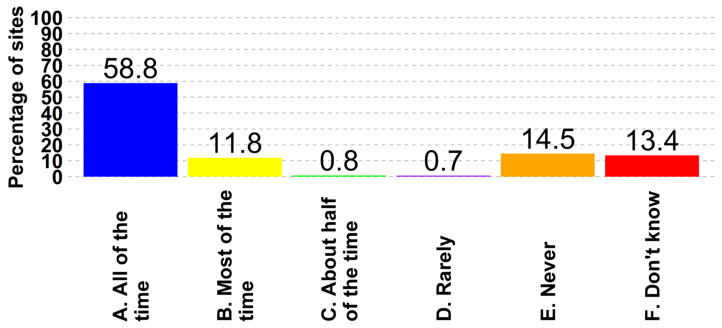
Frequency that feed supplier(s) have held imported feed ingredients to reduce disease transmission risk.

There is variability in the swine industry in regard to the usage of feed mitigants, where 4% (487 of 12,172) of all sites stated they always use feed mitigants, while 62% (7486 of 12,172) said they never did (Figure 8). Nineteen percent of boar studs (12 of 63) and approximately seventeen percent of breeding herds (209 of 1262) reported the usage of feed mitigants all the time. The variability continued in regard to holding times, with 59% (7157 of 12,172) of the respondents having reported holding imported feed ingredients specifically to reduce the transmission risk, and 15% (1765 of 12,172) said they never did. (Figure 9). Within this collection of participant survey data, there was no information gathered regarding specific conditions of ingredient holding, including holding time or temperature, nor was there further information about the use of feed mitigants, including specific products or inclusion levels.

**Figure 10:**
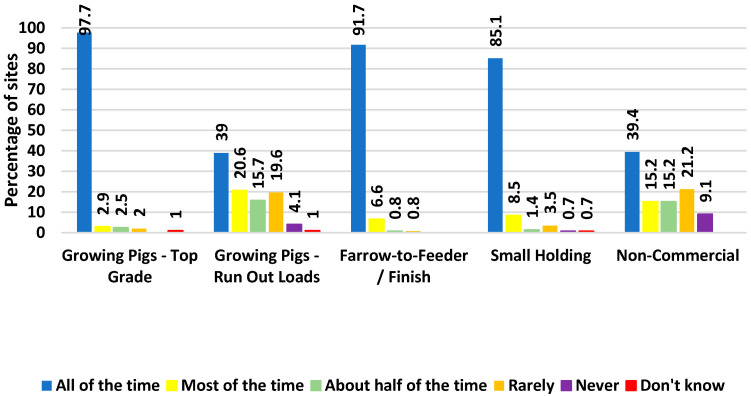
Frequency of transport trailers reported to have been washed before returning to the point of concentration broken down by type of production site.

Related to transport biosecurity, 98.4% (62 of 63) of boar studs, 99.7% of breeding herds (1258 of 1262), and 91.7% (9676 of 10,552) of growing pigs’ top-grade loads reported that the trailers were washed before returning to the point of concentration (e.g., slaughter plant and cull sow buying station). Top-graded pigs are the first loads removed from the site, with the pigs that first reach marketing criteria and usually take place from one to a few weeks before the site is emptied, i.e., loading of run-out loads. Run-out loads and non-commercial sites have a wider dispersion of responses (Figure 10).

## 4. Discussion

This study presents a synopsis of the US industry’s implementation of the current practices of biosecurity that address major risks for disease introduction. Overall, there is limited quantitative information on biosecurity practices being implemented across swine production sites in the US. Having an understanding of biosecurity practices is helpful to design better disease control programs, such as the US SHIP, to ultimately prevent the introduction and spread of diseases. As part of developing a voluntary producer-led government-endorsed disease certification program in the US, named US SHIP, a set of questions were required for the participants to enroll their sites in the program. Biosecurity survey responses provided baseline information on certain biosecurity practices with the goal of identifying and quantifying areas for biosecurity improvement to prevent the introduction of FADs and the spread of endemic diseases.

In this study, a biosecurity practice survey questionnaire was distributed to pork producers through the US SHIP enrollment program, starting in January of 2022 and going through 1 March 2024, and it has been filled out for 12,195 pork production sites over multiple farm types, across 31 states. The captured survey responses reflect the diversity in biosecurity practices of the US swine industry and provide a snapshot of biosecurity practices in different production types, presenting an opportunity to assess and enhance its biosecurity measures over time to mitigate the threats posed by FADs. Commercial breeding herds and growing pig sites were the majority of the sites responding to the US SHIP biosecurity survey.

Production owners and site supervisors can use the findings of this survey to better prepare themselves to battle the FAD threat and work within the US SHIP to develop practices to address site-specific concerns and industry-wide shortfalls. An example of an action item generated by such information is the US SHIP biosecurity working group evaluating what mitigation strategies should be implemented in indoor and outdoor pigs to help decrease the risk posed by feral pigs and tick vectors in FAD transmission [32,33,34]. Findings, advancements, and novel approaches can be shared with producer delegates at US SHIP House of Delegates Meetings.

Having measures in place before any FAD introduction could help decrease the risks of foreign disease introduction and reduce the chance of endemic diseases spreading across farms. Every pig in the US is important in matters of FAD defense, and every producer has different challenges but shares the same responsibility. The results of this survey can better help industries and industry regulators’ direct education, effort, and funds to strengthen biosecurity of the industry.

Diseases, especially FADs, are one of the most urgent concerns facing animal production. Countries such as Brazil [35], the Czech Republic [36], and Belgium [37] have been able to eradicate ASF from their borders after an introduction by using strategies and techniques described by the World Organization of Animal Health (WOAH). Efficient biosecurity and biocontainment practices take advantage of strategies and techniques developed through experience and research and proven to be effective in real-word scenarios for reducing the frequency of disease occurrence and spread across farms, regions, countries, and continents.

A multitiered approach is necessary to ensure the continued ability of pork producers to operate in the face of a FAD. The US Federal Government uses international trade agreements, customs, and border inspections to combat the introduction of FADs. Individual sites must also be prepared to prevent and mitigate the introduction of an FAD. This study identified potential areas of improvement by farm type. An example is the difference between incorporation of biosecurity best practices, such as reducing animal outdoor access and controlling visitor entry, where differences between commercial and non-commercial sites are evident. Across all sections of production, there is an opportunity to improve biosecurity practices, such as showering, the tracking of people’s movements, and truck washing and disinfection, to minimize the risk of disease spread across sites. Even though breeding herds and growing sites are well-positioned to have SPS plans in place, 8.5% of breeding herds and 13.4% of growing sites are still without a biosecurity plan in place that leaves another practical opportunity for improvement.

With 18% of sites lacking entry requirements and 30% requiring only change in clothes, there exists an avoidable risk that can be effectively managed though the implementation of more stringent entry protocols. Entry protocols and sign-in books are practices requiring discipline to follow procedures and could be implemented irrespective of the current infrastructure present on the farm. Continuous training of farm personnel in following biosecurity and biocontainment practices is necessary to keep the discussions and reminders on top of the head of farm employees. Other biosecurity and biocontainment practices, such as promoting the utilization of feed mitigants (currently not employed by 61.5% of respondents) and adhering to proper holding times for imported feed ingredients (practiced by only 58.8% of respondents) are more costly but could significantly reduce the potential risks for FADs through the entry of contaminated feed. The ability of viral particles to maintain infectivity in feed has had real world impacts and is cited as the cause for the introduction of Senecavirus A into a previously naïve country [38]. Specifically for holding imported ingredients, there remains significant variation in how this practice is applied at the industry level, which warrants further investigation to generate a standardized baseline program for the US swine industry to generate a meaningful reduction in FAD introduction and dissemination risk. Another very costly procedure is the implementation of cleaning and disinfection procedures for hauling trucks. Extreme cold weather temperatures during winter pose a challenge in implementing these practices, requiring expensive infrastructure facilities to perform the procedure and treat the effluents.

In general, this study found that breeding herd sites routinely apply more biosecurity practices than other types of sites. Non-commercial sites were less likely to adopt any one specific biosecurity element discussed. This is relevant, as the swine industry is not stove-piped into vacuums, and each industry sector impacts the other and the overall security of the US swine industry [39,40]. Biosecurity improvements in one site or industry type can contribute collectively contain the introduction, establishment, and further spread of threats that affect the overall health of the swine industry.

One limitation of this descriptive study is that all results are self-reported by US SHIP participants. While the producers report their standards, the actual workday practices have not been evaluated. Together, this information provides a baseline understanding of the biosecurity practices in the US swine industry within the participant pool of US SHIP-enrolled sites. The reporting of current stages of biosecurity practices implemented across US SHIP participant farms generates the basic foundation that can be used in future assessments for comparisons of the advancements made across participant sites.

The US SHIP program is a collaboration of swine producers and allied industry, state, and federal partners aimed at safeguarding the US swine industry, and it helps support business continuity in the event of an FAD introduction into the US. This baseline biosecurity through continued producer enrollment and engagement and the evaluation of current practices, as performed in this report, will serve as a means to generate meaningful improvement in biosecurity practices and enhance FAD preparedness and continue to decrease the detrimental impacts of endemic pathogens. The current reporting of biosecurity practices allows for information sharing and the future benchmarking of practices implemented and progress towards improving biosecurity and biocontainment measures to protect the US swine production.

## 5. Conclusions

In conclusion, this initial cross-sectional investigation sheds light on the biosecurity practices within the United States’ swine industry, providing insights derived from the data collected through the US Swine Health Improvement Plan (US SHIP) enrollment program. The findings underscore the industry’s diverse approaches to biosecurity. The establishment of US SHIP as a voluntary initiative demonstrates a collaborative effort among industry stakeholders, state, and federal entities to enhance disease preparedness. Multiple opportunities to improve the current level of biosecurity throughout all facets of the swine industry were identified and here reported. These findings offer a foundation for ongoing assessments, fostering continuous improvement in biosecurity measures and ultimately fortifying the industry’s ability to combat the introduction and spread of diseases, both foreign and endemic. As the swine sector evolves, the knowledge gained from this study is pivotal for shaping targeted strategies to mitigate risks, promote sustainable practices, and contribute to the resilience of the US swine industry against emerging threats.

## Figures and Tables

**Table 1:** US SHIP enrolment biosecurity survey questions.

Questions by Site Type		Response Format
1—Sites that have completed the Secure Pork Supply plans		Response entered in percentile form from 0 to 100.
2—Sites were animals have access to the outdoors.		
3—Sites that have perimeter fences.		
4—Sites that use the following as their primary means of dead disposal	(a) Rendering	Response entered in percentile form from 0 to 100. The total should be equal to 100.
	(b) Non-rendering	
	(c) A combination of rendering and non-rendering	
5—Sites whose requirements for people to enter the farm most closely resembles on of the following	(a) No specific requirements	Response entered in percentile form from 0 to 100. The total should be equal to 100.
	(b) Only visitors are required to change into clean or site-specific clothes and footwear without a requirement to shower in	
	(c) Everyone changes into clean or site-specific clothes and footwear, but are not required to shower in	
	(d) Everyone showers and changes into clean or site-specific clothes and footwear	
6—Do visitors sign a logbook?		Lickert Scale, with responses on a 1–5 scale: (a) All of the time; (b) Most of the time; (c) About half of the time; (d) Rarely; (e) Never; (f) Don’t know.
7—During the last 12 months, how often have you used the following ingredients in rations?	Plasma	
	Meat and bone meal	
	Feed mitigants to reduce the disease transmission risk	
	Uncooked food scraps	
	Regulated (cooked) garbage	
8—During the last 12 months, how often have your feed suppliers held the imported feed ingredients to reduce disease transmission risk?		
9—Have trailers been washed and disinfected since last returning from a point of concentration when being used to pick up animals from the site? *		

* For growing sites, the survey question on trailer wash was divided into two questions: (i) The first asked if the trucks being used to transport the first group of pigs (first cut) to market were washed and disinfected. (ii) The second asked if, when the remaining pigs in the barn were moved to slaughter (run out loads), the trucks were washed and disinfected. The movement of pigs between the first cut and when the barn is emptied occurs within the time frame of a couple of weeks.

**Table 2:** US SHIP enrolment biosecurity survey site-type definitions.

Site Types	Definition
Boar stud	Production site with mature boars that distribute semen to other production sites
Breeding herd	Production site with breeding females and house ≥1000 breeder or feeder swine
Growing pig	Production site with ≥1000 feeder swine
Farrow-to-feeder /finish	Production site with breeding females, grow feeder swine for purposes other than breeding stock replacement for this particular farm site, and house ≥1000 breeder or feeder swine
Small holding	Production sites with ≥100 and ≤1000 breeder or feeder swine
Non-commercial	Production sites with <100 pigs

## Data Availability

All the relevant collected data are provided in this manuscript. Individual enrolment survey responses are confidential and cannot be shared.

## Supplementary Materials

The following supporting information can be downloaded at: https://www.mdpi.com/article/10.3390/ani14071134/s1, File S1: US SHIP Biosecurity Enrollment Survey.
