# Supplementary material for: Biosecurity Insights from the United States Swine Health Improvement Plan: Analyzing Data to Enhance Industry Practices
Source: Animals (Basel). 2024 Apr 8;14(7):1134. doi: 10.3390/ani14071134 (PMC11011101; doi:10.3390/ani14071134)
Supplement: Supplementary file 1 [file animals-14-01134-s001.zip › animals-2948465-supplementary.pdf]

US SHIP Biosecurity Enrollment Survey

Start of Block: Default Question Block

Q0 **US Swine Health Improvement Plan (US SHIP) Biosecurity Enrollment Survey**
Participants are to complete a biosecurity survey at the time of enrollment for each state in which they have participating premises located. The survey responses are to be specific and limited to the participating premises located within each respective state. Completion of the biosecurity survey is required for certification.

The US SHIP participant will need to refer to their US SHIP enrollment information and know how many premises (by production site type) are located within each state.

The survey aims to capture some very basic information concerning biosecurity practices. Information from this survey is to provide a quantitative assessment of the current standards of practice across a broad spectrum of US pork industry participants. Results will serve to help to provide insight towards consideration of additional US SHIP biosecurity-related program standards in the future. Results will be summarized in aggregate / anonymized form only.

This biosecurity survey is to be completed by the US SHIP Program Participant (Swine Owner) or the US SHIP Program Participant's designee that has a direct working knowledge of the participating premises.

Survey participants are being asked to simply complete the survey to the best of their ability.

Responses received will be reviewed and confirmed by the US SHIP Official State Agency.

Page Break

Q1 Participants are to enroll sites with each US SHIP Official State Agency in which they have participating facilities located.

If you have sites located in more than one state, **please fill out one survey for each state**.

The survey responses are to be specific and limited to the participating premises located within each respective state.

Please select the **STATE** for which you are filling out this survey for.

▼ Alabama (1) ... Wyoming (50)

Page Break

Q2 **US SHIP Participant and Point of Contact**
(Used by US SHIP Official State Agency in Confirming Receipt Of Survey Results)

- ○ Swine Owner (US SHIP Participant / Entity): (1) ____
- ○ Address of Swine Owner (US SHIP Participant / Entity): (2) ____
- ○ Phone # of Swine Owner (US SHIP Participant / Entity): (3) ____
- ○ Email of Swine Owner (US SHIP Participant / Entity): (9) ____
- ○ Name of person to be the initial preferred point of contact: (10) ____
- ○ Best Contact Email: (11) ____
- ○ Best Contact Phone Number: (12) ____

Q3 **Please enter the total number of sites for each respective site type enrolled in US SHIP in (Selected State).**

○ BOAR STUD: *Production site with mature boars that distribute semen to other production sites* (1) ____

○ BREEDING HERD: *Production site with ≥ 1,000 breeding females. (e.g., breed-to-wean, breeding/gestation or farrowing only, with or without on-site gilt isolation/grow-out).* (2) ____

○ GROWING PIG: *Production site with ≥ 1,000 feeder swine (nursery, grower, or finisher).* (3) ____

○ FARROW-TO-FEEDER/FINISH: *Production site with breeding females and grow feeder swine for purposes other than breeding stock replacement for this particular farm site, and house ≥ 1,000 breeder or feeder swine.* (9) ____

○ SMALL HOLDING: *Production sites with ≥ 100 and < 1,000 total breeder or feeder swine.* (10) ____

○ NON-COMMERCIAL: *Production sites with < 100 pigs (e.g., exhibition, niche, hobby)* (11) ____

○ PACKING PLANT: *A facility that slaughters pigs* (12) ____

○ LIVE ANIMAL MARKETING OPERATIONS: *A dealer with a livestock yard/buying station (facility) that markets > 100 swine/week for resale of such swine to slaughter facilities* (17) ____

Page Break

End of Block: Default Question Block

Start of Block: Boar Stud

Q4 **The following questions apply only to your *BOAR STUD* site(s).**

You are filling this survey for (Provided number of sites) *BOAR STUD* site(s) in the state of (Selected State).

Q5 1. Enter percentage of *BOAR STUD* site(s) that have completed the Secure Pork Supply Plans (SPS).
Note: *A SPS plan is considered complete when the SPS documents are readily available for review by the competent authority.*

(Enter percentage as a whole number, 0 to 100)

____

Q6 2. Enter percentage of *BOAR STUD* site(s) where animals have access to the outdoors.

(Enter percentage as a whole number, 0 to 100)

____

Q7 3. Enter percentage of *BOAR STUD* site(s) that have perimeter fences.

(Enter percentage as a whole number, 0 to 100)

____

Q8 4. Enter percentage of *BOAR STUD* site(s) that use the following as their primary means of dead disposal?

(Enter percentage as a whole number, 0 to 100. Total should be equal to 100)

	Percentage of sites (1)
a. Rendering (1)	
b. Non-Rendering Option (On-Site or Regional Composting, Burial, or Incineration) (2)	
c. Routinely use a combination of Rendering and Non-Rendering Options (3)	
Total	

Q9 5. Enter percentage of *BOAR STUD* site(s) whose requirements for people to enter the farm most closely resemble one of the following:

(Enter percentage as a whole number, 0 to 100. Total should be equal to 100)

	Percentage of sites (1)
a. No specific requirements for primary caretakers or visitors to change into clean or site specific clothes and footwear. (1)	
b. Only visitors to the farm site are required to change into clean or site specific clothes and footwear, with or without a requirement to shower-in. (2)	
c. Everyone changes into clean or site specific clothes and footwear, but are not required to shower-in. (3)	
d. Everyone showers-in and change into clean or site specific clothes and footwear. (8)	
Total	

Q10 6. For your ***BOAR STUD*** site(s), visitors sign a log-in book prior to entering the site.

	All of the time (2)	Most of the time (3)	About half of the time (8)	Rarely (9)	Never (10)	Don't know (11)
(15)	○	○	○	○	○	○

Q11 7. Regarding feed ingredients and risk management, during the last 12 months, how often have you used the following ingredients in feed rations for BOAR STUD site(s)?

	All of the time (2)	Most of the time (3)	About half of the time (8)	Rarely (9)	Never (10)	Don't know (11)
a. Plasma (7)	○	○	○	○	○	○
b. Meat & Bone Meal (8)	○	○	○	○	○	○
c. Feed mitigants to reduce disease transmission risk (13)	○	○	○	○	○	○
d. Uncooked food scraps (11)	○	○	○	○	○	○
e. Regulated (cooked) garbage (12)	○	○	○	○	○	○

Q12 8. During the last 12 months, how often have your feed supplier(s) held imported feed ingredients to reduce disease transmission risk to BOAR STUD site(s)?

	All of the time (2)	Most of the time (3)	About half of the time (8)	Rarely (9)	Never (10)	Don't know (11)
(7)	○	○	○	○	○	○

Q13 9. When transporting animals from *BOAR STUD* site(s) to terminal points of concentration (i.e., buying station, cull market, or slaughter facility).

	All of the time (2)	Most of the time (3)	About half of the time (4)	Rarely (5)	Never (6)	Don't know (7)
Trailers being used to pick-up animals from BOAR STUD site(s) have been washed and disinfected since last returning from a point of concentration. (7)	○	○	○	○	○	○

End of Block: Boar Stud

Start of Block: Breeding Herds

Q14 **The following questions apply only to your *BREEDING HERD* site(s).**

You are filling this survey for (Provided number of sites) *BREEDING HERD* site(s) in the state of (Selected State).

Q15 1. Enter percentage of *BREEDING HERD* site(s) that have completed the Secure Pork Supply Plans (SPS).
Note: *A SPS plan is considered complete when the SPS documents are readily available for review by the competent authority.*

(Enter percentage as a whole number, 0 to 100)

____

Q16 2. Enter percentage of *BREEDING HERD* site(s) where animals have access to the outdoors.

(Enter percentage as a whole number, 0 to 100)

____

Q17 3. Enter percentage of *BREEDING HERD* site(s) that have perimeter fences.

(Enter percentage as a whole number, 0 to 100)

____

Q18 4. Enter percentage of *BREEDING HERD* site(s) that use the following as their primary means of dead disposal.

(Enter percentage as a whole number, 0 to 100. Total should be equal to 100)

	Percentage of sites (1)
a. Rendering (1)	
b. Non-Rendering Option (On-Site or Regional Composting, Burial, or Incineration) (2)	
c. Routinely use a combination of Rendering and Non-Rendering Options (3)	
Total	

Q19 5. Enter percentage of *BREEDING HERD* site(s) whose requirements for people to enter the farm most closely resemble one of the following:

(Enter percentage as a whole number, 0 to 100. Total should be equal to 100)

	Percentage of sites (1)
a. No specific requirements for primary caretakers or visitors to change into clean or site specific clothes and footwear. (1)	
b. Only visitors to the farm site are required to change into clean or site specific clothes and footwear, with or without a requirement to shower-in. (2)	
c. Everyone changes into clean or site specific clothes and footwear, but are not required to shower-in. (3)	
d. Everyone showers-in and change into clean or site specific clothes and footwear (8)	
Total	

Q20 6. For your *BREEDING HERD* site(s), visitors sign a log-in book prior to entering the site.

	All of the time (2)	Most of the time (3)	About half of the time (8)	Rarely (9)	Never (10)	Don't know (11)
(15)	○	○	○	○	○	○

Q21 7. Regarding feed ingredients and risk management, during the last 12 months, how often have you used the following ingredients in feed rations for BREEDING HERD site(s)?

	All of the time (2)	Most of the time (3)	About half of the time (8)	Rarely (9)	Never (10)	Don't know (11)
a. Plasma (7)						
b. Meat & Bone Meal (8)						
c. Feed mitigants to reduce disease transmission risk (13)						
d. Uncooked food scraps (11)						
e. Regulated (cooked) garbage (12)						

Q22 8. During the last 12 months, how often have your feed supplier(s) held imported feed ingredients to reduce disease transmission risk to BREEDING HERD site(s)?

	All of the time (2)	Most of the time (3)	About half of the time (8)	Rarely (9)	Never (10)	Don't know (11)
(7)						

Q23 9. When transporting animals from *__BREEDING HERD__* site(s) to terminal points of concentration (i.e., buying station, cull market, or slaughter facility).

	All of the time (2)	Most of the time (3)	About half of the time (4)	Rarely (5)	Never (6)	Don't know (7)
Trailers being used to pick-up animals from BREEDING HERD site(s) have been washed and disinfected since last returning from a point of concentration. (7)	○	○	○	○	○	○

End of Block: Breeding Herds

Start of Block: Growing sites

Q24 **The following questions apply only to your *__GROWING PIG__* site(s).**

You are filling this survey for (Provided number of sites) *__GROWING PIG__* site(s) in the state of (Selected State).

Q25 1. Enter percentage of *__GROWING PIG__* site(s) that have completed the Secure Pork Supply Plans (SPS).
Note: *A SPS plan is considered complete when the SPS documents are readily available for review by the competent authority.*

(Enter percentage as a whole number, 0 to 100)

____

Q26 2. Enter percentage of *__GROWING PIG__* site(s) where animals have access to the outdoors.

(Enter percentage as a whole number, 0 to 100)

____

Q27 3. Enter percentage of *GROWING PIG* site(s) that have perimeter fences.

(Enter percentage as a whole number, 0 to 100)

____

Q28 4. Enter percentage of *GROWING PIG* site(s) that use the following as their primary means of dead disposal?

(Enter percentage as a whole number, 0 to 100. Total should be equal to 100)

	Percentage of sites (1)
a. Rendering (1)	
b. Non-Rendering Option (On-Site or Regional Composting, Burial, or Incineration) (2)	
c. Routinely use a combination of Rendering and Non-Rendering Options (3)	
Total	

Q29 5. Enter percentage of *GROWING PIG* site(s) whose requirements for people to enter the farm most closely resemble one of the following:

(Enter percentage as a whole number, 0 to 100. Total should be equal to 100)

	Percentage of sites (1)
a. No specific requirements for primary caretakers or visitors to change into clean or site specific clothes and footwear. (1)	
b. Only visitors to the farm site are required to change into clean or site specific clothes and footwear, with or without a requirement to shower-in. (2)	
c. Everyone changes into clean or site specific clothes and footwear but are not required to shower-in (3)	
d. Everyone showers-in and change into clean or site specific clothes and footwear (8)	
Total	

Q30 6. For your *GROWING PIG* site(s), visitors sign a log-in book prior to entering the site.

	All of the time (2)	Most of the time (3)	About half of the time (8)	Rarely (9)	Never (10)	Don't know (11)
15 (15)	o	o	o	o	o	o

Q31 7. Regarding feed ingredients and risk management, during the last 12 months, how often have you used the following ingredients in feed rations for GROWING PIG site(s)?

	All of the time (2)	Most of the time (3)	About half of the time (8)	Rarely (9)	Never (10)	Don't know (11)
a. Plasma (7)	○	○	○	○	○	○
b. Meat & Bone Meal (8)	○	○	○	○	○	○
c. Feed mitigants to reduce disease transmission risk (13)	○	○	○	○	○	○
d. Uncooked food scraps (11)	○	○	○	○	○	○
e. Regulated (cooked) garbage (12)	○	○	○	○	○	○

Q32 8. During the last 12 months, how often have your feed supplier(s) held imported feed ingredients to reduce disease transmission risk to GROWING PIG site(s)?

	All of the time (2)	Most of the time (3)	About half of the time (8)	Rarely (9)	Never (10)	Don't know (11)
(7)	○	○	○	○	○	○

Q33 9. When transporting top (graded) loads from *GROWING PIG* site(s) to terminal points of concentration (i.e., slaughter facility, buying station, cull market, etc.):

Note: Top (graded) loads are defined as loads where remaining pigs are left on the site (premises) for a period longer than the next 7 days.

	All of the time (2)	Most of the time (3)	About half of the time (4)	Rarely (5)	Never (6)	Don't know (7)
Trailers being used to pick-up animals from GROWING PIG site(s) have been washed and disinfected since last returning from a point of concentration. (7)	o	o	o	o	o	o

Q34 10. When transporting run out loads from *GROWING PIG* site(s) to terminal points of concentration (i.e., slaughter facility, buying station, cull market, etc.):

Note: Run out loads are defined as loads where all the remaining pigs left on site (premises) will be moved to a terminal point of concentration within 7 days.

	All of the time (2)	Most of the time (3)	About half of the time (4)	Rarely (5)	Never (6)	Don't know (7)
Trailers being used to pick-up animals from GROWING PIG site(s) have been washed and disinfected since last returning from a point of concentration. (7)	o	o	o	o	o	o

End of Block: Growing sites

Start of Block: Farrow-to-Feeder/Finish

Q35 **The following questions apply only to your *FARROW-TO-FEEDER/FINISH* site(s).**

You are filling this survey for (Provided number of sites) *FARROW-TO-FEEDER/FINISH* site(s) in the state of (Selected State).

Q36 1. Enter percentage of *FARROW-TO-FEEDER/FINISH* site(s) that have completed the Secure Pork Supply Plans (SPS).
Note: *A SPS plan is considered complete when the SPS documents are readily available for review by the competent authority.*

(Enter percentage as a whole number, 0 to 100)

____

Q37 2. Enter percentage of *FARROW-TO-FEEDER/FINISH* site(s) where animals have access to the outdoors.

(Enter percentage as a whole number, 0 to 100)

____

Q38 3. Enter percentage of *FARROW-TO-FEEDER/FINISH* site(s) that have perimeter fences.

(Enter percentage as a whole number, 0 to 100)

____

Q39 4. Enter percentage of *FARROW-TO-FEEDER/FINISH* site(s) that use the following as their primary means of dead disposal?

(Enter percentage as a whole number, 0 to 100. Total should be equal to 100)

	Percentage of sites (1)
a. Rendering (1)	
b. Non-Rendering Option (On-Site or Regional Composting, Burial, or Incineration) (2)	
c. Routinely use a combination of Rendering and Non-Rendering Options (3)	
Total	

Q40 5. Enter percentage of *FARROW-TO-FEEDER/FINISH* site(s) whose requirements for people to enter the farm most closely resemble one of the following:

(Enter percentage as a whole number, 0 to 100. Total should be equal to 100)

	Percentage of sites (1)
a. No specific requirements for primary caretakers or visitors to change into clean or site specific clothes and footwear. (1)	
b. Only visitors to the farm site are required to change into clean or site specific clothes and footwear, with or without a requirement to shower-in (2)	
c. Everyone changes into clean or site specific clothes and footwear but are not required to shower-in (3)	
d. Everyone showers-in and change into clean or site specific clothes and footwear (8)	
Total	

Q41 6. For your *FARROW-TO-FEEDER/FINISH* site(s), visitors sign a log-in book prior to entering the site.

	All of the time (2)	Most of the time (3)	About half of the time (8)	Rarely (9)	Never (10)	Don't know (11)
(15)	○	○	○	○	○	○

Q42 7. Regarding feed ingredients and risk management, during the last 12 months, how often have you used the following ingredients in feed rations for FARROW-TO-FEEDER/FINISH site(s)?

	All of the time (2)	Most of the time (3)	About half of the time (8)	Rarely (9)	Never (10)	Don't know (11)
a. Plasma (7)	o	o	o	o	o	o
b. Meat & Bone Meal (8)	o	o	o	o	o	o
c. Feed mitigants to reduce disease transmission risk (13)	o	o	o	o	o	o
d. Uncooked food scraps (11)	o	o	o	o	o	o
e. Regulated (cooked) garbage (12)	o	o	o	o	o	o

Q43 8. During the last 12 months, how often have your feed supplier(s) held imported feed ingredients to reduce disease transmission risk to FARROW-TO-FEEDER/FINISH site(s)?

	All of the time (2)	Most of the time (3)	About half of the time (8)	Rarely (9)	Never (10)	Don`t know (11)
(7)	o	o	o	o	o	o

Q44 9. When transporting animals from *FARROW-TO-FEEDER/FINISH* site(s) to terminal points of concentration (i.e., buying station, cull market, or slaughter facility).

	All of the time (2)	Most of the time (3)	About half of the time (4)	Rarely (5)	Never (6)	Don`t know (7)
Trailers being used to pick-up animals from FARROW-TO-FEEDER/FINISH site(s) have been washed and disinfected since last returning from a point of concentration. (7)	o	o	o	o	o	o

End of Block: Farrow-to-Feeder/Finish

Start of Block: Small holding

Q45 **The following questions apply only to your *SMALL HOLDING* site(s).**

You are filling this survey for (Provided number of sites) *SMALL HOLDING* site(s) in the state of (Selected State).

Q46 1. Enter percentage of *SMALL HOLDING* site(s) that have completed the Secure Pork Supply Plans (SPS).
Note: *A SPS plan is considered complete when the SPS documents are readily available for review by the competent authority.*

(Enter percentage as a whole number, 0 to 100)

____

Q47 2. Enter percentage of *SMALL HOLDING* site(s) where animals have access to the outdoors.

(Enter percentage as a whole number, 0 to 100)

____

Q48 3. Enter percentage of *SMALL HOLDING* site(s) that have perimeter fences.

(Enter percentage as a whole number, 0 to 100)

____

Q49 4. Enter percentage of *SMALL HOLDING* site(s) that use the following as their primary means of dead disposal?

(Enter percentage as a whole number, 0 to 100. Total should be equal to 100)

	Percentage of sites (1)
a. Rendering (1)	
b. Non-Rendering Option (On-Site or Regional Composting, Burial, or Incineration) (2)	
c. Routinely use a combination of Rendering and Non-Rendering Options (3)	
Total	

Q50 5. Enter percentage of <u>*SMALL HOLDING*</u> site(s) whose requirements for people to enter the farm most closely resemble one of the following:

(Enter percentage as a whole number, 0 to 100. Total should be equal to 100)

	Percentage of sites (1)
a. No specific requirements for primary caretakers or visitors to change into clean or site specific clothes and footwear. (1)	
b. Only visitors to the farm site are required to change into clean or site specific clothes and footwear, with or without a requirement to shower-in (2)	
c. Everyone changes into clean or site specific clothes and footwear but are not required to shower-in (3)	
d. Everyone showers-in and change into clean or site specific clothes and footwear (8)	
Total	

Q51 6. For your *SMALL HOLDING* site(s), visitors sign a log-in book prior to entering the site.

	All of the time (2)	Most of the time (3)	About half of the time (8)	Rarely (9)	Never (10)	Don't know (11)
15 (15)	○	○	○	○	○	○

Q52 7. Regarding feed ingredients and risk management, during the last 12 months, how often have you used the following ingredients in feed rations for SMALL HOLDING site(s)?

	All of the time (2)	Most of the time (3)	About half of the time (8)	Rarely (9)	Never (10)	Don't know (11)
a. Plasma (7)	○	○	○	○	○	○
b. Meat & Bone Meal (8)	○	○	○	○	○	○
c. Feed mitigants to reduce disease transmission risk (13)	○	○	○	○	○	○
d. Uncooked food scraps (11)	○	○	○	○	○	○
e. Regulated (cooked) garbage (12)	○	○	○	○	○	○

Q53 8. During the last 12 months, how often have your feed supplier(s) held imported feed ingredients to reduce disease transmission risk to SMALL HOLDING site(s)?

	All of the time (2)	Most of the time (3)	About half of the time (8)	Rarely (9)	Never (10)	Don't know (11)
(7)	○	○	○	○	○	○

Q54 9. When transporting animals from *SMALL HOLDING* site(s) to terminal points of concentration (i.e., buying station, cull market, or slaughter facility).

	All of the time (2)	Most of the time (3)	About half of the time (4)	Rarely (5)	Never (6)	Don`t know (7)
Trailers being used to pick-up animals from SMALL HOLDING site(s) have been washed and disinfected since last returning from a point of concentration. (7)	○	○	○	○	○	○

End of Block: Small holding

Start of Block: Non-comercial

Q55 **The following questions apply only to your *NON-COMMERCIAL* site(s).**

You are filling this survey for (Provided number of sites) NON-COMMERCIAL site(s) in the state of (Selected State).

Q56 1. Enter percentage of *NON-COMMERCIAL* site(s) that have completed the Secure Pork Supply Plans (SPS).
Note: *A SPS plan is considered complete when the SPS documents are readily available for review by the competent authority.*

(Enter percentage as a whole number, 0 to 100)

____

Q57 2. Enter percentage of *NON-COMMERCIAL* site(s) where animals have access to the outdoors.

(Enter percentage as a whole number, 0 to 100)

____

Q58 3. Enter percentage of *NON-COMMERCIAL* site(s) that have perimeter fences.

(Enter percentage as a whole number, 0 to 100)

____

Q59 4. Enter percentage of *NON-COMMERCIAL* site(s) that use the following as their primary means of dead disposal?

(Enter percentage as a whole number, 0 to 100. Total should be equal to 100)

	Percentage of sites (1)
a. Rendering (1)	
b. Non-Rendering Option (On-Site or Regional Composting, Burial, or Incineration) (2)	
c. Routinely use a combination of Rendering and Non-Rendering Options (3)	
Total	

Q60 5. Enter percentage of *NON-COMMERCIAL* site(s) whose requirements for people to enter the farm most closely resemble one of the following:

(Enter percentage as a whole number, 0 to 100. Total should be equal to 100)

	Percentage of sites (1)
a. No specific requirements for primary caretakers or visitors to change into clean or site specific clothes and footwear (1)	
b. Only visitors to the farm site are required to change into clean or site specific clothes and footwear, with or without a requirement to shower-in. (2)	
c. Everyone changes into clean or site specific clothes and footwear but are not required to shower-in. (3)	
d. Everyone showers-in and change into clean or site specific clothes and footwear (8)	
Total	

Q61 6. For your *NON-COMMERCIAL* site(s), visitors sign a log-in book prior to entering the site.

	All of the time (2)	Most of the time (3)	About half of the time (8)	Rarely (9)	Never (10)	Don't know (11)
(15)	○	○	○	○	○	○

Q62 7. Regarding feed ingredients and risk management, during the last 12 months, how often have you used the following ingredients in feed rations for NON-COMMERCIAL site(s)?

	All of the time (2)	Most of the time (3)	About half of the time (8)	Rarely (9)	Never (10)	Don't know (11)
a. Plasma (7)	○	○	○	○	○	○
b. Meat & Bone Meal (8)	○	○	○	○	○	○
c. Feed mitigants to reduce disease transmission risk (13)	○	○	○	○	○	○
d. Uncooked food scraps (11)	○	○	○	○	○	○
e. Regulated (cooked) garbage (12)	○	○	○	○	○	○

Q63 8. During the last 12 months, how often have your feed supplier(s) held imported feed ingredients to reduce disease transmission risk to NON-COMMERCIAL site(s)?

	All of the time (2)	Most of the time (3)	About half of the time (8)	Rarely (9)	Never (10)	Don't know (11)
(7)	○	○	○	○	○	○

Q64 9. When transporting animals from *NON-COMMERCIAL* site(s) to terminal points of concentration (i.e., buying station, cull market, or slaughter facility).

	All of the time (2)	Most of the time (3)	About half of the time (4)	Rarely (5)	Never (6)	Don't know (7)
Trailers being used to pick-up animals from NON-COMMERCIAL site(s) have been washed and disinfected since last returning from a point of concentration. (7)	○	○	○	○	○	○

End of Block: Non-comercial

Start of Block: Live Animal Marketing Operation

Q67 **The following questions apply only to your *LIVE ANIMAL MARKETING OPERATION*.**

You are filling this survey for (Provided number of sites) LIVE ANIMAL MARKETING OPERATION(s) in the state of (Selected State).

Q68 1. Enter percentage of *LIVE ANIMAL MARKETING OPERATION*(s) where animals have access to the outdoors.

(Enter percentage as a whole number, 0 to 100)

____

Q69 2. Enter percentage of *LIVE ANIMAL MARKETING OPERATION*(s) that have perimeter fences.

(Enter percentage as a whole number, 0 to 100)

____

Q70 3. Enter percentage of *LIVE ANIMAL MARKETING OPERATION* (s) that use the following as their primary means of dead disposal?

(Enter percentage as a whole number, 0 to 100. Total should be equal to 100)

	Percentage of sites (1)
a. Rendering (1)	
b. Non-Rendering Option (On-Site or Regional Composting, Burial, or Incineration) (2)	
c. Routinely use a combination of Rendering and Non-Rendering Options (3)	
Total	

Q71 4. Enter percentage of *LIVE ANIMAL MARKETING OPERATION*(s) whose requirements for people to enter the farm most closely resemble one of the following:

(Enter percentage as a whole number, 0 to 100. Total should be equal to 100)

	Percentage of sites (1)
a. No specific requirements for primary caretakers or visitors to change into clean or site specific clothes and footwear. (1)	
b. Only visitors to the farm site are required to change into clean or site specific clothes and footwear, with or without a requirement to shower-in (2)	
c. Everyone changes into clean or site specific clothes and footwear but are not required to shower-in (3)	
d. Everyone showers-in and change into clean or site specific clothes and footwear (8)	
Total	

Q72 5. For your *LIVE ANIMAL MARKETING OPERATION*(s), visitors sign a log-in book prior to entering the site.

	All of the time (2)	Most of the time (3)	About half of the time (8)	Rarely (9)	Never (10)	Don't know (11)
(15)	○	○	○	○	○	○

Q73 6. Regarding feed ingredients and risk management, during the last 12 months, how often have you used the following ingredients in feed rations for LIVE ANIMAL MARKETING OPERATION(s)?

	All of the time (2)	Most of the time (3)	About half of the time (8)	Rarely (9)	Never (10)	Don't know (11)
a. Plasma (7)	○	○	○	○	○	○
b. Meat & Bone Meal (8)	○	○	○	○	○	○
c. Feed mitigants to reduce disease transmission risk (13)	○	○	○	○	○	○
d. Uncooked food scraps (11)	○	○	○	○	○	○
e. Regulated (cooked) garbage (12)	○	○	○	○	○	○

Q74 7. During the last 12 months, how often have your feed supplier(s) held imported feed ingredients to reduce disease transmission risk to <u>LIVE ANIMAL MARKETING OPERATION</u> (s)?

	All of the time (2)	Most of the time (3)	About half of the time (8)	Rarely (9)	Never (10)	Don`t know (11)
(7)	○	○	○	○	○	○

Q75 8. When transporting animals from *LIVE ANIMAL MARKETING OPERATION*(s) to slaughter facility.

	All of the time (2)	Most of the time (3)	About half of the time (4)	Rarely (5)	Never (6)	Don`t know (7)
Trailers being used to pick-up animals from LIVE ANIMAL MARKETING OPERATION (s) have been washed and disinfected since last returning from a slaughter facility. (7)	o	o	o	o	o	o

End of Block: Live Animal Marketing Operation

Start of Block: Block 7

Q76
You have reached the end of the US SHIP Biosecurity Enrollment Survey.

Please click on the arrow below to submit your survey.
Thank you!

End of Block: Block 7
